# Smart mid-infrared metasurface microspectrometer gas sensing system

**DOI:** 10.1038/s41378-024-00697-2

**Published:** 2024-06-07

**Authors:** Jiajun Meng, Sivacarendran Balendhran, Ylias Sabri, Suresh K. Bhargava, Kenneth B. Crozier

**Affiliations:** 1 School of Physics, University of Melbourne, Victoria, Australia; 2 Australian Research Council (ARC) Centre of Excellence for Transformative Meta-Optical Systems (TMOS), University of Melbourne, Victoria, Australia; 3 Centre for Advanced Materials & Industrial Chemistry (CAMIC), STEM college, RMIT University, Victoria, Australia; 4 Department of Electrical and Electronic Engineering, University of Melbourne, Victoria, Australia

**Keywords:** Optical sensors, Micro-optics

## Abstract

Smart, low-cost and portable gas sensors are highly desired due to the importance of air quality monitoring for environmental and defense-related applications. Traditionally, electrochemical and nondispersive infrared (IR) gas sensors are designed to detect a single specific analyte. Although IR spectroscopy-based sensors provide superior performance, their deployment is limited due to their large size and high cost. In this study, a smart, low-cost, multigas sensing system is demonstrated consisting of a mid-infrared microspectrometer and a machine learning algorithm. The microspectrometer is a metasurface filter array integrated with a commercial IR camera that is consumable-free, compact ( ~ 1 cm^3^) and lightweight ( ~ 1 g). The machine learning algorithm is trained to analyze the data from the microspectrometer and predict the gases present. The system detects the greenhouse gases carbon dioxide and methane at concentrations ranging from 10 to 100% with 100% accuracy. It also detects hazardous gases at low concentrations with an accuracy of 98.4%. Ammonia can be detected at a concentration of 100 ppm. Additionally, methyl-ethyl-ketone can be detected at its permissible exposure limit (200 ppm); this concentration is considered low and nonhazardous. This study demonstrates the viability of using machine learning with IR spectroscopy to provide a smart and low-cost multigas sensing platform.

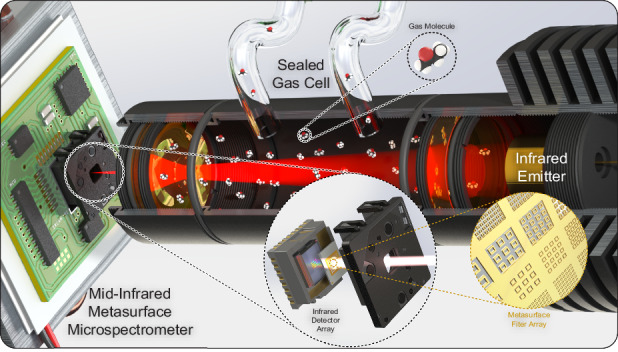

## Introduction

There is currently considerable demand for gas sensing technology due to its numerous applications; these include atmospheric pollution monitoring, the detection of hazardous gas leaks in industry, and the detection of harmful volatile organic compounds in indoor locations^1,2^. Moreover, in recent years, the emergence of the Internet of Things (IoT) has spurred interest in developing smart gas sensor systems. These systems combine sensors with advanced signal processing techniques and machine learning algorithms^3^, enabling the resultant system to perform real-time analysis of the gases present. While much progress has been made, low-cost smart gas sensors that can simultaneously achieve low detection limits and low cross-sensitivity in situations where multiple gases need to be detected have yet to be developed. Current gas sensing techniques can be categorized as follows: electrochemical gas sensors, optical gas sensors, acoustic-based sensors, gas chromatography (GC) sensors and calorimetric-based sensors^1^. Electrochemical-based sensors have been attained using materials that include carbon nanotubes^4^, semiconductor nanowires^5,6^, and 2D materials such as α-MoO_3_, graphene and MXene^7–9^. These sensors have high sensitivity but suffer from limited lifetimes and cross-response issues^10^. Acoustic gas sensors typically detect gases by measuring the ultrasonic wave velocity, attenuation and acoustic impedance^11^; however, these sensors are limited by high power consumption. GC is commonly used for laboratory chemical analysis and has excellent chemical separation performance, with high sensitivity and selectivity^12^. GC systems generally have large footprints and are nonportable; thus, they are unsuitable for use as smart gas sensors. Calorimetric gas sensors detect gases based on differences in their heat of combustion^13^. However, this approach tends to have low sensitivity and selectivity for gas sensing. Optical gas sensing methods include fluorescent chemosensors^14^, nondiffractive infrared (NDIR) sensors^15^, and absorption spectroscopy-based sensors^2^. Fluorescent chemosensors convert chemical stimuli into a detectable fluorescent response. Although this technique requires low power consumption, it faces challenges such as poor reusability and slow response time^16^. Both NDIR and absorption spectroscopy-based gas sensing detect gases based on their mid-infrared (MIR) “fingerprints,” i.e., the unique absorption spectra of chemicals due to the molecular vibrational modes excited by infrared radiation. This approach can provide a fast response, minimal drift, high specificity, long lifetime and robustness to changes in the ambient environment^10^. NDIR is usually implemented by monitoring the intensity of analyte IR absorption at a single (or a few) wavelength(s) achieved by filtering the IR source to match the absorption line(s). The current workhorse tool for IR spectroscopy is the Fourier transform infrared (FTIR) spectrometer. FTIR effectively performs for gas sensing, but its platform is generally large, has high power consumption and is expensive. Thus, the development of gas sensors that use IR spectroscopy as a sensing mechanism with favorable size, weight, power consumption and cost is the topic of this paper.

In this study, we construct a smart optical gas sensing system employing an MIR metasurface microspectrometer (MIMM) to detect multiple hazardous gases down to hundreds of parts per million (ppm) levels. Previous studies have demonstrated infrared microspectrometers using spectral filtering elements; these include voltage-tunable Fabry‒Pérot (FP) filters^17–20^; compact FTIR interferometer-based systems such as Michelson interferometers^21,22^; lamellar gratings^23^; Mach‒Zehnder interferometers^24^; and other approaches such as single-pixel Hadamard transform spectrometers^25^ and arrayed waveguide gratings^26^. While these methods have been effective, they require complicated fabrication methods and/or experimental setups and have limited operating wavelength ranges, which hinders the development of IoT sensor networks using these techniques. IR microspectrometers can be alternatively constructed using the filter-array-detector-array (FADA) architecture^27^. In this configuration, a spectral filter array is placed on top of a detector array. By performing computational analysis of the detector array output, the input spectrum can be reconstructed^28,29^. For some applications, this step is not necessary; for example, the FADA device can be used for IR-based chemical identification by directly analyzing the detector array output without performing spectral reconstruction. This approach is used in our study. An advantage of the FADA architecture is its simplicity; specifically, the data can be attained by adding a spectral filter array to a detector array such as a microbolometer camera. Furthermore, the resultant systems are generally highly robust because they contain no moving parts. In recent years, there has been much interest in the use of metasurfaces as filters, in part because fabrication usually requires only a single lithography step. Previous studies have demonstrated metasurface spectral filtering using plasmonic gratings and waveguides^30–33^, metal-insulator-metal structures^34–37^, quasi bound-state-in-the-continuum (BIC) resonances^38,39^, and guided mode resonances (GMRs)^40^. Among these methods, gas detection was demonstrated in Refs. ^30,34–36^. Unlike our work, Ref. ^30^ did not demonstrate an integrated system. Refs. ^34,35^ demonstrated an integrated system for gas detection but was limited to the detection of one species (CO_2_). Ref. ^36^ demonstrated multiple gas detection methods with an integrated system comprising pyroelectric detectors with plasmonic metamaterial absorbers. A classical approach was used, where each detector/absorber was tailored to detect a particular gas. Eight different gases could be detected by the eight detectors of the device. This is different from our approach. Our MIMM is a lightweight ( ~ 1 g) and small ( ~ 1 cm^3^) device that consists of a metal nanoantenna metasurface filter array integrated with a compact thermal camera. The filter array has twenty spectral channels that span the broad wavelength range of 6–14 μm. Our device does not target specific gases but is highly versatile. In our previous study^27^, we showed that the same device could be used for liquid chemical detection, ranging from common laboratory chemicals (acetone, methanol, isopropanol, and ethanol) to medications (ibuprofen, aspirin, and acetaminophen) and even foodstuffs (olive oil, vegetable oil, and peanut oil). Unlike Ref. ^36^, our device also does not require specialized fabrication methods and can be readily added to a thermal camera. The gas sensing performed here is much more challenging than in our previous liquid detection study^27^ because the analyte absorption is much weaker. Here, we show that by stabilizing the temperature of the MIMM, we drastically reduce its drift, thereby enabling it to be used for gas sensing. We build a gas sensing system centered around the MIMM and subject the system to various gases. Machine learning classifiers (MLCs) are trained using the data collected for four gas species: carbon dioxide (CO_2_), methane (CH_4_), ammonia (NH_3_) and 2-butanone (methyl-ethyl-ketone, MEK). These gases are diluted with nitrogen at various concentrations to represent different hazardous gas detection scenarios. We show that the trained MLC can identify these gases with very high accuracy. We demonstrate MEK detection at the permissible exposure limit (PEL), which refers to the maximum level of exposure to a hazardous substance that a worker can be exposed to over a given time period (usually 8 h per day) without suffering adverse health effects. Specifically, we show that our system can detect a hazardous gas at a concentration considered to be low and nonhazardous. To the best of our knowledge, this study is the first demonstration of a smart mid-IR multigas sensor based on optical metasurfaces and a machine learning classification model.

This paper is organized as follows. We first provide a detailed description of the design and working principle of the metasurface gas sensor; the sensor consists of the MIMM, a custom-made gas cell, and a temperature controller. Next, we discuss the experimental setup, data acquisition, and training of a machine learning classifier for multigas detection. Finally, we conclude by summarizing our findings and discussing future work on gas sensors with enhanced metasurface designs.

## Results and discussion

**Fig. 1:**
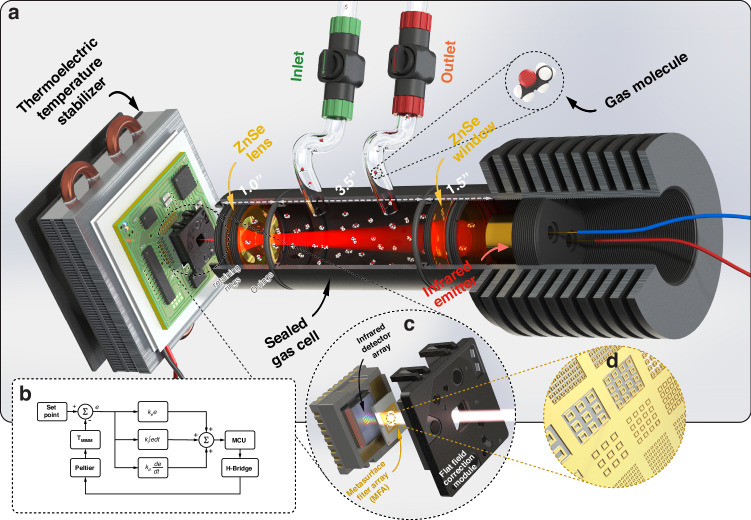
Components and Layout of the Metasurface Gas Sensing System. **a** Schematic of metasurface gas sensing system. The MIMM and infrared emitter are placed on two sides of a custom-made gas cell. The MIMM is mounted on a thermoelectric temperature stabilizer. The gas cell is filled with gas to be sensed. **b** Control loop diagram of the TTS controller. **c** Schematic of the MIMM. Infrared radiation is spectrally filtered by a metasurface array, with transmitted light being absorbed by the pixels of the microbolometer camera. **d** Schematic illustration of a metallic metasurface filter array

**Fig. 2:**
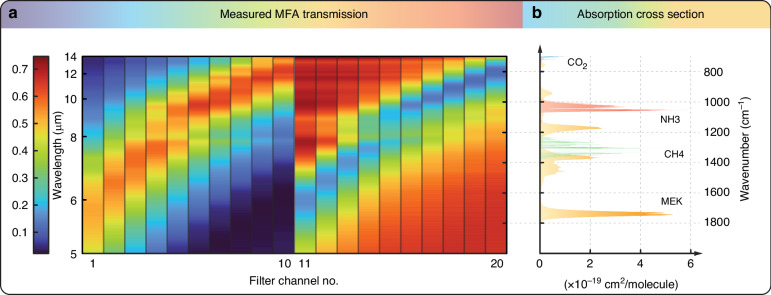
Spectral Characteristics of Metasurface Filters and Gas Absorption. **a** Filter transmission spectra of the fabricated metasurface filters measured using an FTIR spectrometer. The bandstop and bandpass filters have transmittance dips and peaks, respectively, spanning the 6–14 µm (1667–714 cm^−1^) wavelength (wavenumber) range. **b** Absorption cross section spectra of CO_2_, CH_4_, NH_3_ and MEK in the wavelength (wavenumber) range of 5–14.3 µm (2000–700 cm^−1^)

Our metasurface gas sensing system (Fig. 1a) consists of four main parts, i.e., the MIMM, a thermoelectric temperature stabilizer (TTS), a gas cell and a thermal infrared emitter. The MIMM and the thermal emitter are placed on the two sides of the custom-made gas cell. The gas cell is then loaded with the analyte gas to be sensed, and the gas interacts with the infrared radiation. The light transmitted through the cell illuminates the MIMM, striking the twenty-channel metasurface filter array (MFA, Fig. 1d) and the underlying thermal camera. Figure 1c is a schematic illustration of the device. The readout from the MIMM is based on a microbolometer and susceptible to temperature fluctuations that originate from the variations in the ambient temperature in the laboratory and/or from the heat produced by on-board electronics. This susceptibility shows up as a signal drift. Therefore, we introduce a TTS to stabilize the temperature of the MIMM. We characterize the transmission of the MFA using an FTIR microscope (Perkin Elmer Spotlight 200i) before filter bonding. Figure 2a shows that dips and peaks are present in the wavelength range of 6–14 µm for the BSFs and BPFs, respectively, as expected. More details of the MIMM, gas cell and TTS fabrication are provided in the Methods section.

**Fig. 3:**
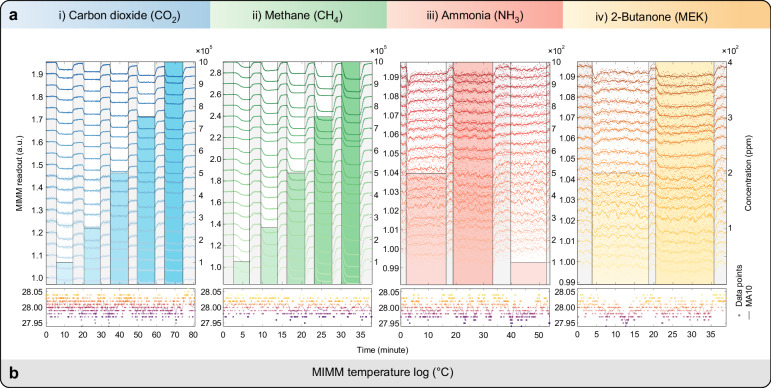
Visualization of MIMM readout data and temperature stability. **a** Acquired MIMM readout data for the four analytes at various concentrations. The data are acquired at concentration steps of [10%, 25%, 50%, 75%, 100%] for CO_2_ and CH_4_, [100, 500, 1000] ppm for NH_3_, and [200, 400] ppm for MEK. The height of the color bars indicates the concentration in the steady state, and the scale is displayed on the right y-axis. The width of the color bars represents the duration of the data acquisition at the specific concentration. Note that for better visibility, the twenty-channel readout values are offset by steps of 0.05, 0.1, 0.005 and 0.005 per channel in the plots for CO_2_, CH_4_, NH_3_ and MEK, respectively. **b** Recorded MIMM temperatures during data collection. The measured temperatures are almost entirely in the range of 28 ± 0.05 °C

We select four gases to demonstrate multianalyte detection using our metasurface gas sensing system: CO_2_, CH_4_, NH_3_, and MEK. Figure 2b shows the absorption cross section spectra of the four gases^41–43^ at room temperature and a pressure of one atmosphere in the wavelength (wavenumber) range 5–14.3 µm (2000–700 cm^−1^). CH_4_, NH_3_ and MEK have prominent absorption cross section peaks in the operating wavenumber range of the MIMM. The absorption of CO_2_ is much weaker than that of the other gases, but CO_2_ can still be detected by our system. To acquire data for each gas at different concentrations, we use a gas mixing setup to dilute the gas with a nitrogen (N_2_) carrier gas; N_2_ does not absorb MIR and is chemically inert. For CO_2_ and CH_4_, with gas cylinder concentrations of 100%, their concentration steps are 10, 25, 50, 75 and 100%. NH_3_ and MEK have lower gas cylinder concentrations; the concentration steps for NH_3_ are 100, 500, and 1000 ppm, and the concentration steps for MEK are 200 and 400 ppm. MEK has a recognized PEL of 200 ppm^44^. While the lowest available possible concentrations for CO_2_, CH_4_ and NH_3_ are above the PELs and are thus inapplicable to hazardous gas detection in breathable air, numerous other applications require gas sensing at these concentrations; some examples included for industrial process monitoring and fuel leak monitoring. More details on the data acquisition and processing can be found in the Methods section. All data are acquired beginning with a baseline recording using N_2_ as a reference. The acquired data is normalized to the mean of the baseline values of each MFA channel, as shown in Fig. 3a. For visualization purposes, the twenty-channel readout values are offset by steps of 0.05, 0.1, 0.005 and 0.005 per channel for CO_2_, CH_4_, NH_3_ and MEK, respectively. The MIMM temperature recorded during data collection is also plotted in Fig. 3b. The temperature stabilizes to 28 ± 0.05 °C during the data collection. The line plots show the data as 10-point moving averages; this enables the underlying trend in the channel readouts as the concentration is varied to be observed more clearly. We plot the gas concentration vs. time as a bar graph in Fig. 3a (right-hand axis). For CO_2_ and CH_4_, the readout values decrease as the concentration increases due to IR absorption by these gases. For NH_3_ and MEK, the changes are more subtle, but they are still detectable.

**Fig. 4:**
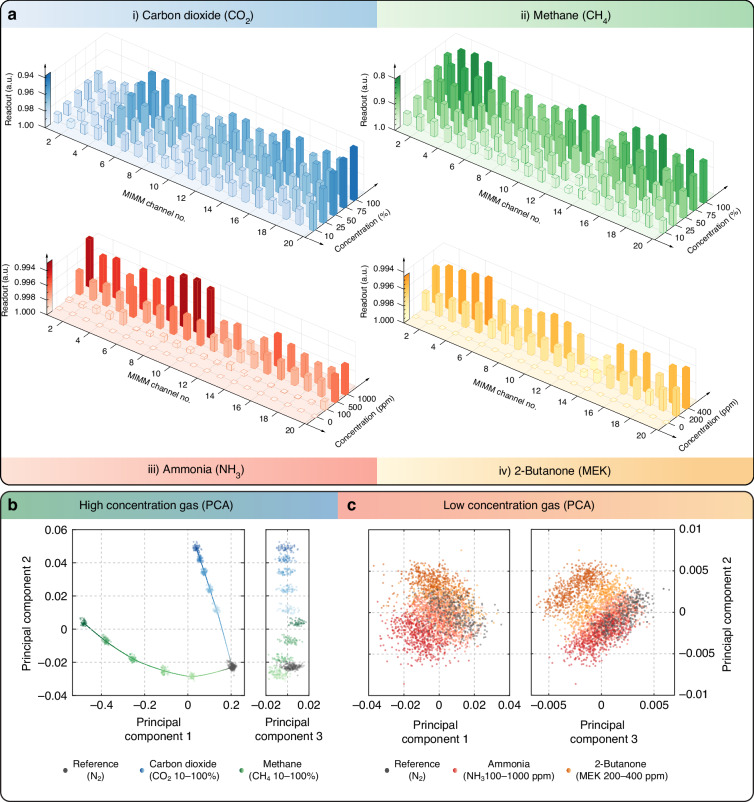
Analysis of MIMM readout data and PCA results for gas detection. **a** 3-D bar plots of the MIMM mean readout values versus concentration and channel number. This shows the discrimination of MFA channels on different gas analytes. **b** Data dimension reduction using PCA on the data of **b** high-concentration gases and **c** low-concentration gases. Only PCs 1–3 are plotted

We plot the data from the gas sensing experiments in Fig. 4a. The data are presented as 3-D bar plots that show the mean acquired readout value for each gas at each concentration under steady-state conditions. This is done by taking data every 5 min after the start of the analyte gas cycle. Each gas has a distinctive pattern; specifically, the different channels of the MIMM discriminate between the different gas analytes. This capability is necessary for the MLC to be able to perform pattern recognition of the readout data and to identify different gases. To further study the distinctiveness of the readout patterns of different gases, we perform principal component analysis (PCA) to reduce the data dimensionality. The visualization of PCA results is shown in Fig. 4b, c, and notably, only principal components (PCs) 1–3 are plotted because they represent most of the explained variance in the data ( > 95%). From Fig. 4b, for CO_2_ and CH_4_, the data from the different species and different concentrations form clusters. For the same type of gas, the datapoints follow a trajectory leading toward the cluster of the “Reference (N_2_)” class as the concentration varies from high to low. Additionally, the paths of these datapoint trajectories are distinctly different for the two gases, which indicates that the trained MLC will exhibit high classification accuracy. We also notice that the data separation in PC3 is not prominent and only accounts for the randomness, i.e., noise, during data acquisition. For the low-concentration gases (NH_3_ and MEK), the PCA results (Fig. 4c) show that the datapoints are clustered much less distinctively due to the much lower signal-to-noise ratio. Nonetheless, as we describe later, the separation of clusters and the concentration trajectories in PC space are sufficient for the MLC to perform accurately. We next describe the training of the MLC for multianalyte detection.

**Fig. 5:**
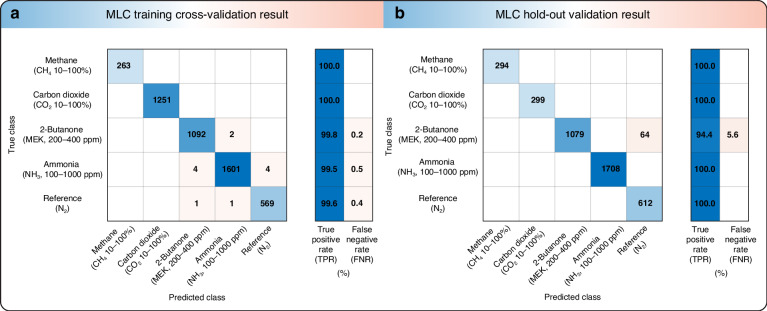
Confusion matrices for MLC validation. MLC **a** training cross-validation results and **b** hold-out validation results, shown as confusion matrices

The MLC algorithms enable the gas sensing system to become “smart,” i.e., to determine the gas present directly from the readout data from the MIMM without performing any spectral reconstruction. We use a support vector machine (SVM) algorithm with quadratic kernels to train the MLC. This method has proven to be very effective in previous studies^27,45^. The trained SVM classifier is first cross-validated within the training dataset to prevent overfitting. The result is presented as a confusion matrix, as shown in Fig. 5a. The overall classification accuracy is 99.75%. All misclassifications are among those of low-concentration gases and the “Reference N_2_” class. To further validate the efficacy of the model, we acquire another dataset two days later for hold-out validation; specifically, we use the trained SVM classifier to perform classification on unseen data. The confusion matrix of the classification results confirms that our model can still identify data points with very high accuracy, and the “2-butanone (MEK, 200–400 ppm)” class is the only classification error and was misclassified as “Reference N_2._” Based on the cross-validation results, we might have anticipated a higher misclassification rate for the “Ammonia (NH_3_, 100–1000 ppm)” class than for the “2-Butanone (MEK, 200–400 ppm)” class for hold-out validation. We attribute this to drift in the system between the training and validation steps, and this can be understood as follows. First, the baseline of the readout values drifts in the acquired hold-out validation dataset. This drift occurs mainly because the source emission spectrum is dependent on the ambient temperature and because the IR emitter temperature is not controlled. Second, even though the use of TTS assists in maintaining a relatively consistent readout stream, the system remains vulnerable to sudden temperature variations that the temperature stabilizer may not always be able to counteract promptly. These abrupt temperature changes can also lead to undesired data drift. We anticipate that obtaining additional data under a variety of environmental conditions can help to address this problem. These data would provide a more precise understanding of the fluctuation in readout values for all analyte gases. Despite the presence of the drift, the overall classification accuracy is 98.40%. All misclassifications appear between MEK and reference classes, i.e., 94.40% classification accuracy for MEK at concentrations down to 200 ppm, i.e., the PEL level of MEK. These results confirm that the MIMM can be used for hazardous gas detection. Although the demonstrated detection of NH_3_, CO_2_ and CH_4_ do not occur at their respective PELs, good performance will likely be obtained even at lower concentrations because at the current tested concentrations, the performance is near perfect. In addition, there are many other applications (e.g., industrial process monitoring and mining safety^46^) which require gas detection at higher concentrations.

## Conclusion

Smart gas sensors have attracted much research interest in recent years to meet the growing demand for applications in industrial manufacturing, agriculture, smart homes, and environmental monitoring. In addition, with the rise of IoT technology, there is a trend toward the deployment of a network of lightweight, small footprint and low-cost sensors. In this study, we design and develop a smart gas sensing system using a mid-infrared metasurface microspectrometer (MIMM) and a machine learning classifier for the detection of multiple hazardous gas compositions. The MIMM consists of a metasurface spectral filter array integrated with a microbolometer camera that is attached to a temperature stabilizer to reduce the readout drift. Its operating wavelength is 7–14 μm (1428–714 cm^−1^), which falls within the MIR “fingerprint” region. We use a gas mixing setup to subject the sensing system to a combination of various gases and acquire a dataset for the machine learning classifier training. The validation results show high accuracy in identifying analytes from a group of gases, including MEK, diluted with N_2_ to its PEL. We anticipate that spectral filters with narrower linewidths will enable higher sensing performance and mixture sensing. Candidates for future work include quasi-BIC structures^38,47^, GMR filters^40^, and FP cavities^48^.

## Methods

### MIMM fabrication

The MIMM is a modified compact thermal camera (FLIR Lepton v2.5) with an integrated metasurface infrared filter chip. The thermal camera has a microbolometer array containing 80 × 60 pixels that are responsive in the wavelength range of 7–14 µm^49^. We have provided a detailed report of the process used to produce the MIMM in our previous work^23^ and thus describe it only briefly here. The spectral response of the filters is determined by the geometry (i.e., dimensions) of the metasurface patterns. The latter are square rings of gold for bandstop filters (BSFs) and square ring-shaped openings in a gold film for bandpass filters (BPFs), as shown in Fig. 1d. The metasurface filters are designed to exhibit spectral features spanning 7–14 µm (1429–714 cm^−1^). Optical microscope photographs and scanning electron micrographs of the fabricated MFA can be found in the Supplementary Information (Figure S2). After its nanofabrication, the MFA chip is glued to the microbolometer array using a die bonder (Finetech Fineplacer Lambda).

### Gas cell

The custom-made gas cell comprises three lens tubes (Ø1” diameter). An inlet and an outlet to the gas cell are fabricated by plugging two polyurethane tubes into holes (Ø6 mm) drilled on the middle lens tube. The IR source is a high-power infrared emitter that generates an optical power of 320 mW and has an aperture of Ø1/2”. The emission is from nanostructured metal rods and has a spectrum close to that of an ideal blackbody^50^. The emitter package consists of a BaF_2_ window soldered to a gold reflective collimator and is filled with nitrogen gas by the manufacturer to lengthen its lifespan. The IR emitter is threaded to a heatsink and mounted onto the lens tube. The same lens tube is also equipped with a zinc selenide (ZnSe) IR window via internal threading. We used an additional lens tube that houses a ZnSe lens (Thorlabs AL72512-E3) to loosely focus the transmitted IR light onto the active region of the MIMM. ZnSe optical elements have antireflection coatings that enable higher transmission in the wavelength band of interest, i.e., the spectral range over which the microbolometer is responsive (7–14 µm or 1428–714 cm^−1^). The gas cell has an optical path length of approximately 12.5 cm. The assembly is hermetically sealed, as shown in Fig. 1a (apart from the gas inlet and outlet), using retaining rings, rubber O-ring gaskets and polytetrafluoroethylene thread seal tape (for the IR optics and the junctions between the lens tubes, not shown in Fig. 1a).

### TTS

The TTS is a Peltier heat pump driven by an electronic circuit (H-bridge) that can provide a load current of alternating polarity, enabling switching between cooling and heating modes. A heatsink is mounted on one side of the Peltier heat pump, while the other side is thermally coupled to the MIMM. The H-bridge circuit is controlled by a microcontroller (Arduino Micro), which generates pulse width modulation (PWM) signals to either cool or heat the MIMM device. We use a proportional-integral-derivative (PID) feedback loop in MATLAB (R2022b) interfaced with both the microcontroller and the internal MIMM temperature sensor. The PID coefficients are heuristically tuned to improve the control stability and minimize the steady-state error, as shown in the control loop diagram of Fig. 1b. The set point is the desired “bias” temperature for the MIMM, and the controller constantly monitors the measured temperature (T_MIMM_) and performs correction. By doing so, we achieve a stabilized temperature of 28 °C with a temperature swing of ±0.1 °C and a standard deviation of the temperature error of ~0.03 °C. A photograph of the complete system is provided in the Supplementary Information (Figure S1).

### Data acquisition and MLC training

The mixing setup consists of source gas cylinders, pressure regulators and mass flow controllers (MFCs). The use of MFCs is critical because they are calibrated to accurately control the volumetric (and mass) flow of gas species, regardless of any pressure and temperature fluctuations. The cylinder concentrations for the analyte gases are as follows: 100% for CO_2_, 100% for CH_4_, 1000 ppm for NH_3_ in N_2_ and 400 ppm for MEK in N_2_. These values represent the upper limits for concentration in our experiments. The lower limit of the diluted concentration is determined by both the mass flow rate resolution and the maximum allowable mass flow rate of the MFCs. In our case, the maximum mixing ratio (flow rate in mL/min) between N_2_ and the analyte gas is 180:20, i.e., the lowest possible concentration limits are 10% CO_2_, 10% CH_4_, 100 ppm NH_3_ and 40 ppm MEK. The output of MFCs produces a constant 200 sccm flow of blended gas mixture that passes through the gas cell via the inlet and outlet. The outlet exhausts to the fume hood in which the system is located. The training of the MLC is performed as follows. For each analyte gas, we sample the readout data from the MIMM every five seconds at different concentrations, including in the case of no analyte gas (i.e., only N_2_ is present). Each readout entry (provided to the MLC) is a 1×20 vector labeled with the corresponding analyte name, concentration, timestamp, and T_MIMM_. The setpoint of the T_MIMM_ is 28 °C and the T_MIMM_ is stabilized by the TTS. We change the mixing ratio between the two MFC channels to increase the concentration step by step. Each step is followed by purging with N_2_ to remove the residual analyte gas in the cell. In addition, before we start the acquisition of the next analyte gas, we precondition the analyte gas MFC by flowing 200 standard cubic centimeters per minute (sccm) of new target gas through it. During this phase, the output gas flow from this MFC is not mixed with any N_2_ and is directed to the fume hood vent. In addition, the gas cell is purged with N_2_ to restore the baseline readout values. All data acquisition, processing, classifier training and validation steps are performed using MATLAB (R2022b) with the image acquisition toolbox with the support package for the OS generic video interface and the statistics and machine learning toolbox. More details of the data processing and statistics are available in the Supplementary Information (Figure S3–6).

### Supplementary information

Supplementary Information
