# Supplementary material for: Smart mid-infrared metasurface microspectrometer gas sensing system
Source: Microsyst Nanoeng. 2024 Jun 7;10:74. doi: 10.1038/s41378-024-00697-2 (PMC11156923; doi:10.1038/s41378-024-00697-2)
Supplement: Supplementary file 1 — Supplementary Information [file 41378_2024_697_MOESM1_ESM.docx]

**Supplementary information**

**Smart Mid-Infrared Metasurface Microspectrometer Gas Sensing System**

Jiajun Meng^1,2*^, Sivacarendran Balendhran^1^, Ylias M. Sabri^3^, Suresh K. Bhargava^3^, and Kenneth B. Crozier^1,2,4*^

^1^ School of Physics, University of Melbourne, Victoria 3010, Australia

^2^ Australian Research Council (ARC) Centre of Excellence for Transformative Meta-Optical Systems (TMOS), University of Melbourne, Victoria 3010, Australia

^3^ Centre for Advanced Materials & Industrial Chemistry (CAMIC), School of Science, Engineering and Health, RMIT University, Victoria 3000, Australia

^4^ Department of Electrical and Electronic Engineering, University of Melbourne, Victoria 3010, Australia

* Author e-mail address: jiajun.meng@unimelb.edu.au, kenneth.crozier@unimelb.edu.au

**1. Gas sensing system**


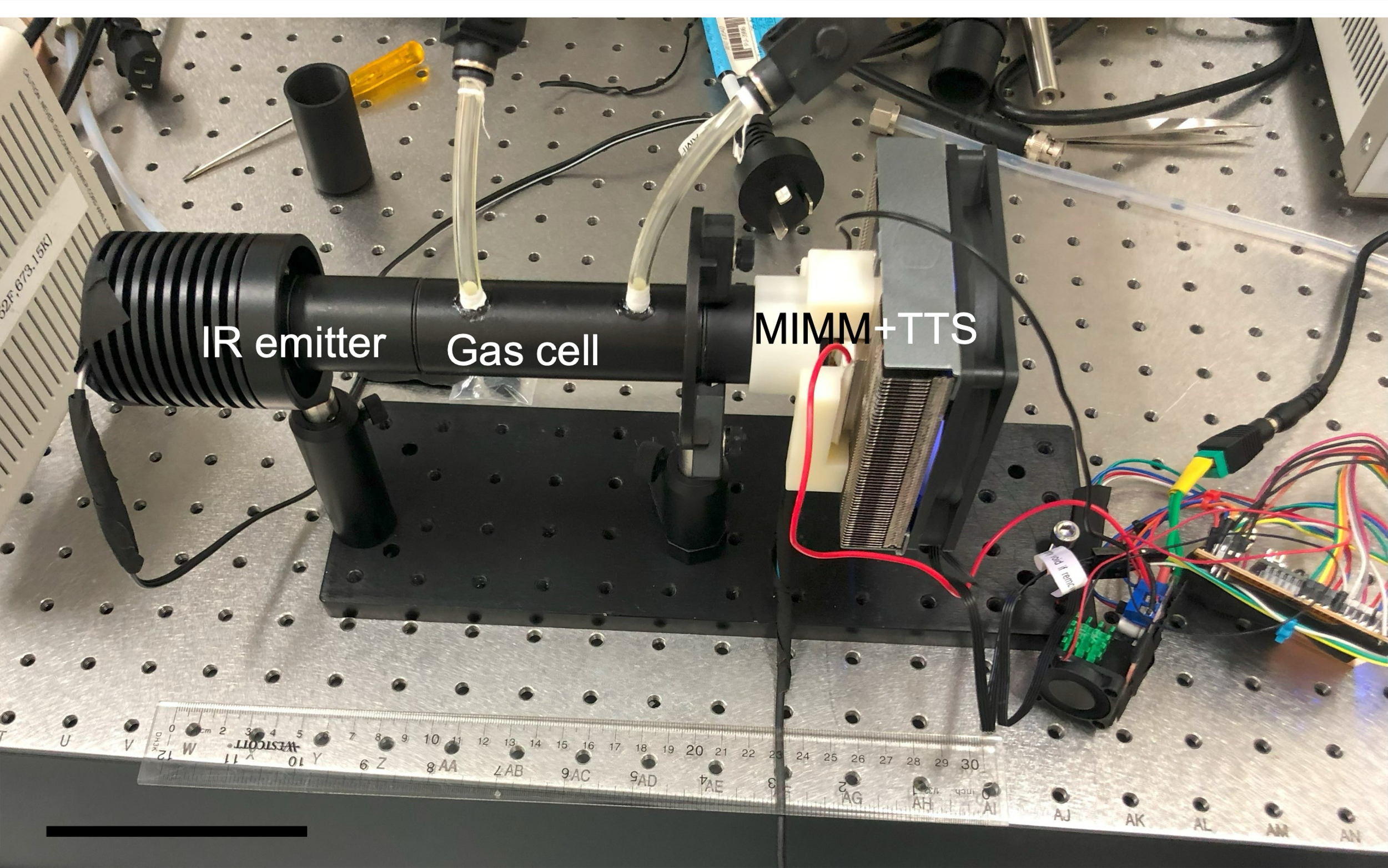


**Figure S1**. (a) Photo of gas sensing system. Scale bar is 10 cm. Electronics on right are microcontroller and H-bridge power supply for Peltier heat pump.

**2. Optical microscope and SEM images of the MFA**


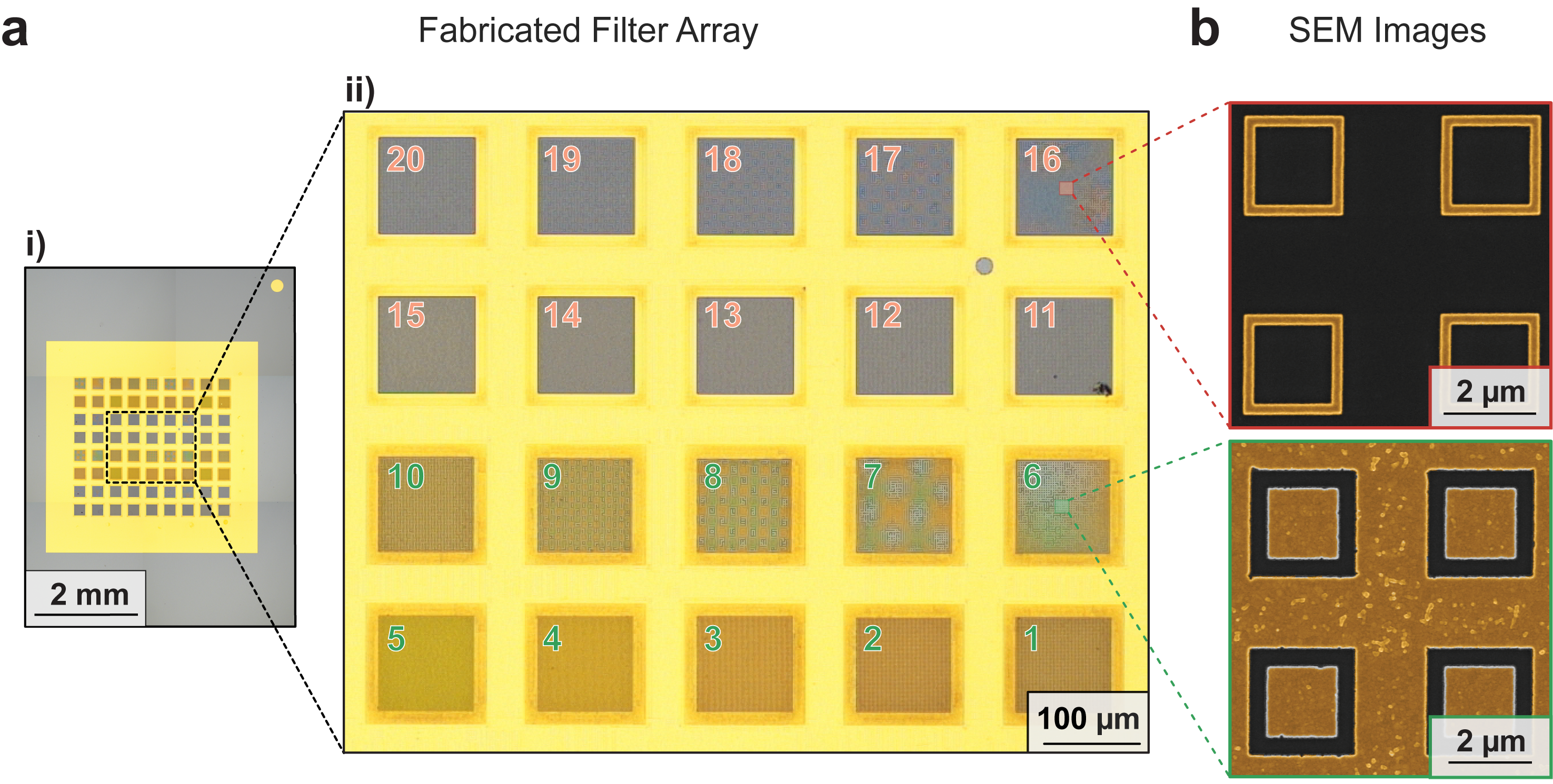


**Figure S2**. (a) (i) Optical microscope image of fabricated MFA chip. (ii) Zoomed view of center 20 filter channels. Filter numbers are shown in top left corners. (b) SEM images of selected bandstop (top, channel no. 16) and bandpass (bottom, channel no. 6) filters. SEM images of fabricated filter array, false-colored for visualization.

**3. Data processing and statistics**


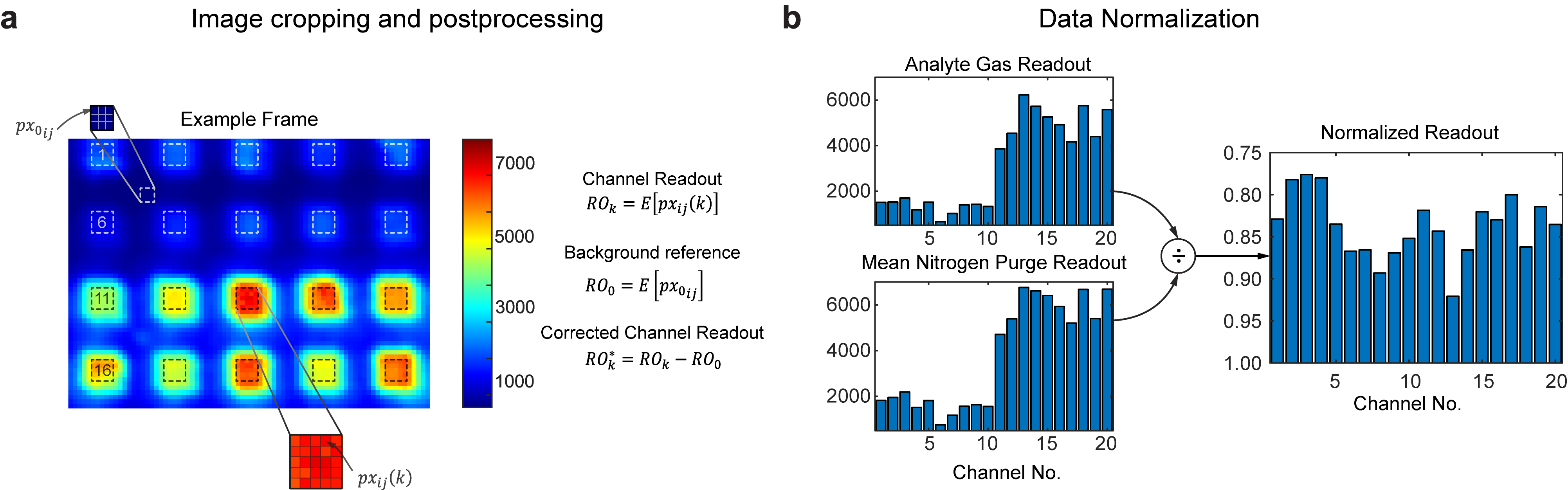


**Figure S3**. Image-to-readout conversion (a) Image cropping and post processing. Example frame is an analyte frame for 100% methane. (b) Data normalization

The acquired raw data is a series of cropped regions of interest (ROI) including all 20 filter channels and an additional background reference area for correction. The pixel values in each ROI are averaged to produce the raw channel readout RO and then we use the background reference readout for correction. The corrected readouts are further normalized using averaged nitrogen purge readout data to obtain the normalized readouts which are used in the machine learning model training.


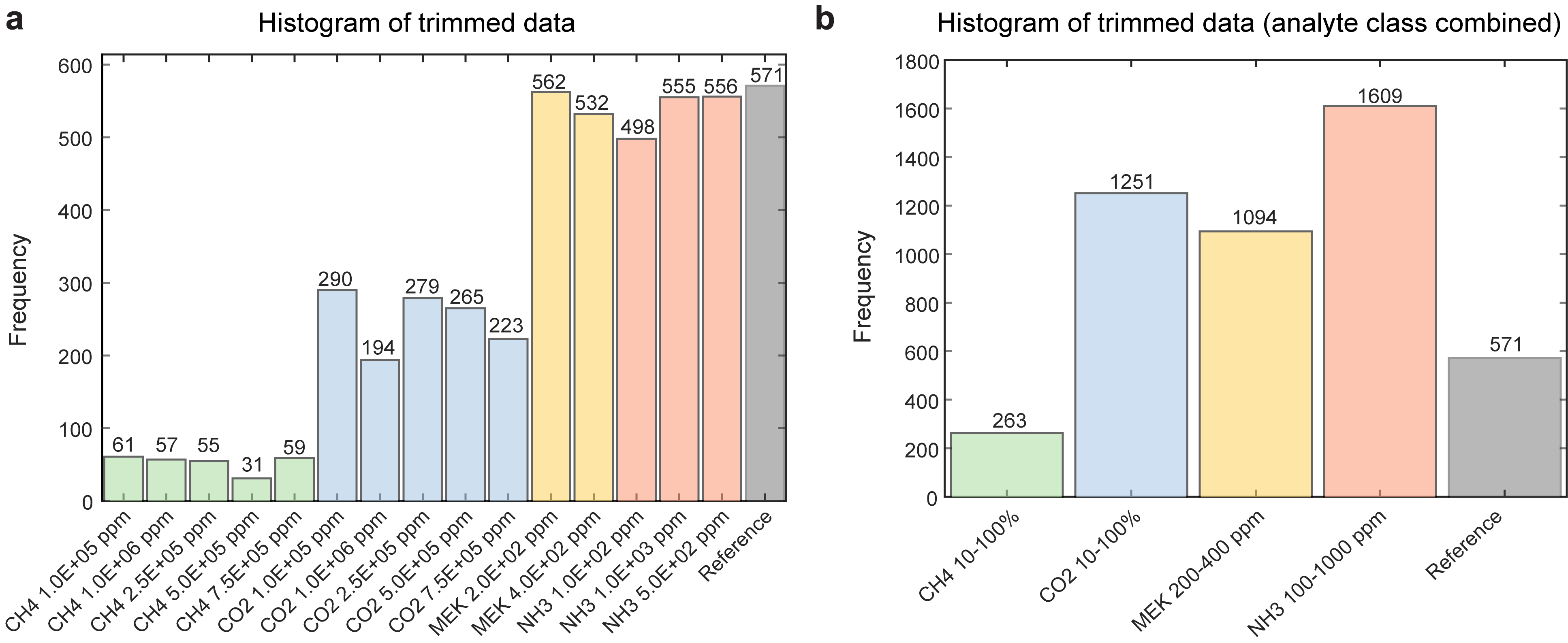


**Figure S4**. (a) Histograms of trimmed data. (b) Histograms of trimmed data after combining the analyte classes.

Data is further trimmed, which is done by picking out steady state data as stated in the main text. Figure S4a is a histogram of trimmed data, plotted for each analyte type at a specific concentration. Figure S4b shows a histogram of trimmed data, plotted for each analyte type at a range of concentrations. We acquired far fewer data points for methane as our supply was limited. We nonetheless find that the number of acquired data points for methane is sufficient for machine learning model training, partly because at the concentrations studied, the signals measured by the sensor vary in a pronounced manner with concentration. This can also be seen in the errorbar plots as shown in Figure S5. It can be seen that the deviation in data is much less for methane. The system noise performance can also be observed from the errorbar plots. As expected, the noise level is much more significant for low concentration analytes than the high concentration ones. The signal to noise ratio (SNR) of channel k can be defined as:

$SNR_{k}=\frac{1-\mu\left( RO_{k} \right)}{\sigma\left( RO_{k} \right)} (1)$

Note that here the system “signal” is defined as the change in normalized readout values compared to nitrogen reference. The calculated SNR is shown as Figure S6. It is quite clear that the SNR is high when analyte concentration is high. In addition, distinct patterns can also be observed for different analytes due to the wavelength selectivity of different channels and how those channels interact with the transmitted IR light from a certain analyte. Some of the SNR values are negative due to the drift in the baseline. This is only seen in lower concentration cases.

**
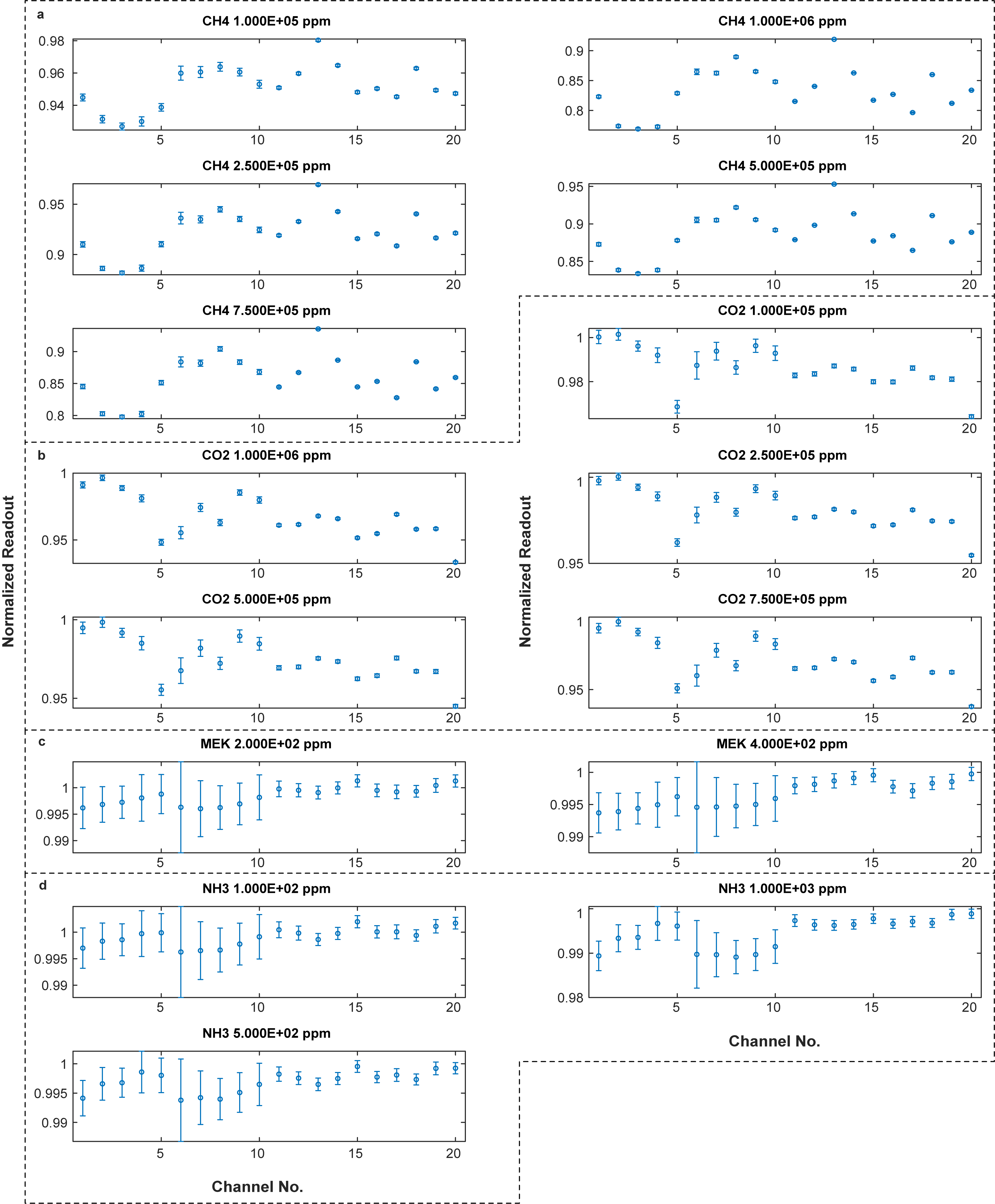
**

**Figure S5**. Errorbar plots of normalized readout for (a) methane, (b) carbon dioxide, (c) 2-butanone and (d) ammonia. Lengths of errorbars represent twice the standard deviation.


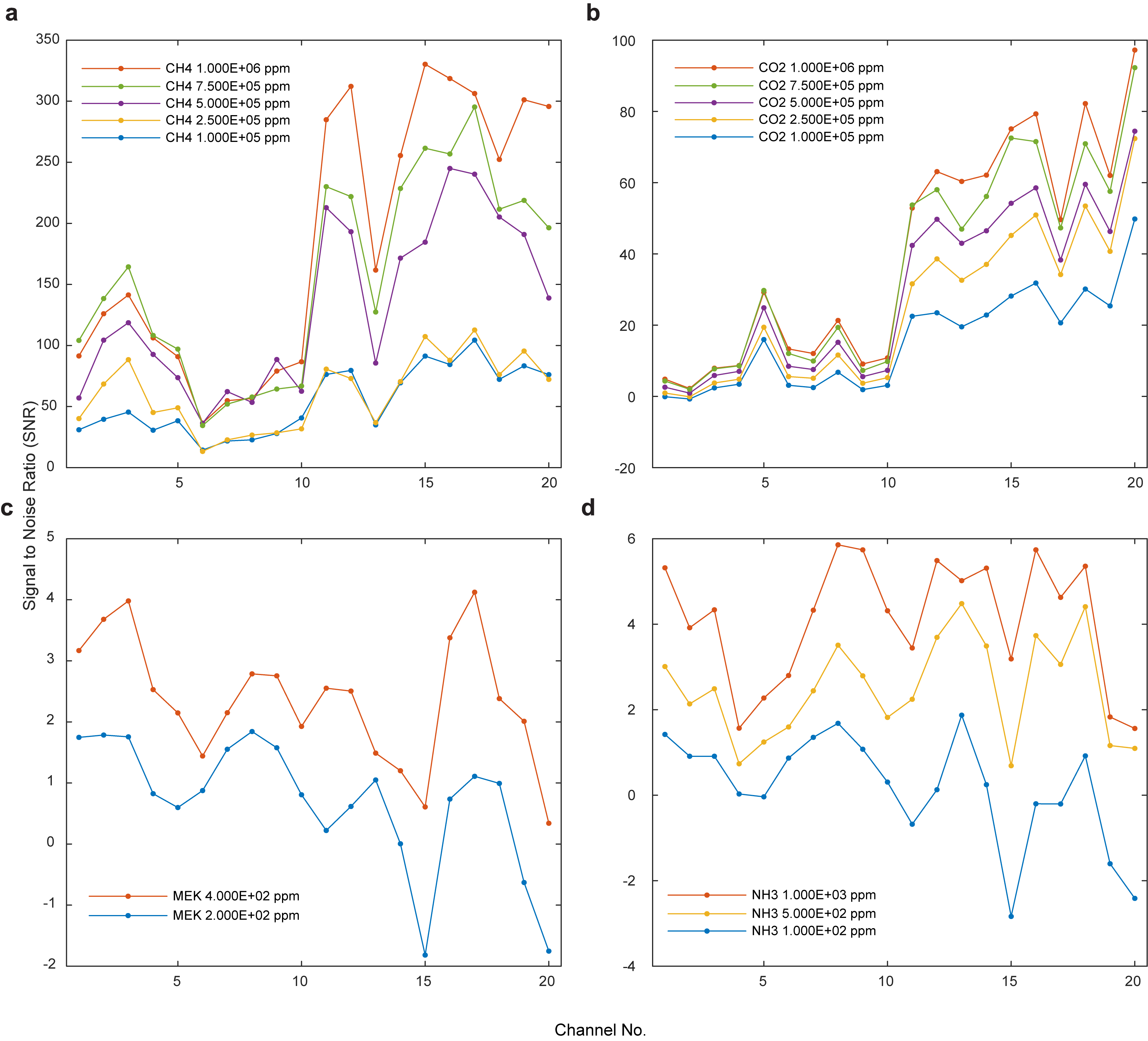


**Figure S6**. Signal to noise ratio of each channel for (a) methane, (b) carbon dioxide, (c) butanone and (d) ammonia.
